# Three-Dimensional Visualization and Imaging of the Entry Tear and Intimal Flap of Aortic Dissection Using CT Virtual Intravascular Endoscopy

**DOI:** 10.1371/journal.pone.0164750

**Published:** 2016-10-19

**Authors:** Yafei Qi, Xiaoyuan Ma, Gang Li, Xiangxing Ma, Qing Wang, Dexin Yu

**Affiliations:** 1 Qilu Hospital of Shandong University, Jinan, Shandong Province, China; 2 Radiology Department, Jiaotong Hospital of Shandong Province, Jinan, Shandong Province, China; 3 Radiology Department, Qilu Hospital of Shandong University, Jinan, Shandong Province, China; Medical University Innsbruck, AUSTRIA

## Abstract

**Aims:**

Conventional computed tomography (CT) approaches provides limited visualization of the entire endoluminal changes of aortic dissection (AD), which is essential for its treatment. As an important supplement, three-dimensional CT virtual intravascular endoscopy (VIE) can show relevant details. This study aims to determine the value of VIE in displaying the entry tear and intimal flap of AD.

**Methods and Results:**

Among 127 consecutive symptomatic patients with suspected AD who underwent CT angiography (CTA), 84 subjects were confirmed to have AD and were included in the study. Conventional CT and VIE images were observed and evaluated. From the 92 entry tears revealed via conventional CT, 88 (95.7%) tears appeared on VIE with round (n = 26), slit-shaped (n = 9), or irregular (n = 53) shapes, whereas the intimal flaps were sheetlike (n = 34), tubular (n = 34), wavelike (n = 13), or irregular (n = 7) in shape. The VIE also showed the spatial relationship between the torn flap and adjacent structures. Among 58 entry tears with multiple-line type flap shown on conventional CT, 41 (70.7%) appeared with an irregular shape on VIE, whereas among 30 tears with single-line type flap, 17 (56.7%) appeared as round or slit-shaped on VIE. These results demonstrated a significant difference (*P* < 0.05). The poor display of tears on VIE was related to the low CT attenuation values in lumen or in neighboring artifacts (*P* < 0.01).

**Conclusion:**

CT VIE presents the complete configurations and details of the intimal tears and flaps of AD better than conventional CT approaches. Accordingly, it should be recommended as a necessary assessment tool for endovascular therapy and as part of strategy planning in pre-surgical patients.

## Introduction

With the increasing prevalence of minimally invasive treatment approaches for aortic dissection (AD), endovascular stent-graft implantation has become widely accepted as a good alternative to traditional surgical repair [1–3], which requires accurate localization of an intimal tear site [4]. Therefore, the complete visualization of endoluminal details, including abnormal changes in the intimal tear and flap of AD, as well as the relationship between changes and adjacent anatomic structures, is essential for planning and assessing treatment [5,6]. Over the last several years, multi-slice computed tomography (CT) has become a crucial technology in the diagnosis and assessment of AD. This technique exhibits higher reliability and feasibility than magnetic resonance imaging and transesophageal echocardiography [7–9]. Available guidelines for diagnosis are based on conventional CT approaches, which typically include axial imaging supplemented with two-dimensional (2D) or three-dimensional (3D) reconstructions. Such reconstructions consist of multiplanar reformation (MPR), maximum intensity projection (MIP), and volume rendering (VR) [10–12]. In most cases, the obtained images are sufficient for diagnosis and assessment. However, in patients with complicated dissection, these methods provide a limited display of the entire endoluminal structures and changes [10], thereby posing a major challenge in accurate assessment. By contrast, virtual intravascular endoscopy (VIE) overcomes this limitation and exhibits a unique value in the complete visualization of endovascular details [13–15]. To date, however, the clinical value of VIE in the visualization of AD has not yet been given sufficient attention. The present study aims to evaluate the value of VIE in imaging 3D endovascular changes in patients with AD prior to surgical or endovascular procedures, as well as to investigate the factors that influence VIE display.

## Materials and Methods

### Study Population

From August 2008 to December 2011, 127 consecutive symptomatic patients with suspected AD were referred to our hospital for aortic CT angiography (CTA). The patient inclusion criterion was AD confirmed via CTA with good imaging display that would allow analysis and evaluation using a workstation. A total of 84 patients (59 females and 25 males, with ages ranging from 28 years to 79 years, mean age: 55.1 ± 11.7 years) were included in this retrospective study. Among the subjects, 54 cases were caused by high blood pressure, 19 by type 2 diabetes mellitus, 5 by Marfan syndrome, and 1 by trauma. The causes of the other cases were uncertain. The remaining 34 patients were excluded from the study because 29 cases were not AD, whereas the imaging quality for the other 5 cases was extremely poor, which made it difficult to obtain VIE images. The ethics committee of our hospital approved the study, and a written informed consent was obtained from all the subjects.

### CTA Imaging

Electrocardiograph (ECG)-gated-synchronized dual-source CTA (Somatom Definition, Siemens, Germany) was performed with a scanning field that typically covered the entire aorta and pelvic vessels prior to treatment. Automatic bolus triggering scan was initiated when the triggering threshold at the region of interest, which was placed in the proximal descending thoracic aorta, reached the preset value of 120 Hounsfield units (HU). A non-ionic iodine contrast agent (370 mg I/mL) with a total volume of 100 mL was administered into the antecubital vein at a flow rate of 4–6 mL/s using a binocular high-pressure syringe via a 20-gauge intravenous catheter, followed by the subsequent injection of 40–60 mL isotonic saline. Retrospectively, ECG-gated reconstructions were made and analyzed during systole (30%–40%) or diastole (70%–75%). The scanning parameters were as follows: gantry rotation time, 0.33 s; tube potential, 2×100 kVp or 2×120 kVp if BMI > 30; effective tube current, 320–400 mAs depending on patient size; pitch, 1.2; collimation, 32 mm × 0.6 mm; and beam collimation, 64 mm × 0.6 mm. The scanning time was 7–11 s. The effective dose was calculated from the dose length product (DLP) using a conversion coefficient of 0.017 mSvmGy^−1^cm^−1^. The radiation dose per individual measured approximately 27.8 ± 4.3 mSv.

### Reconstruction and Observation of the Images

Axial images with a section thickness of 1 mm in the optimal cardiac phase (diastolic phase in 59 patients and systolic phase in 25) were transferred to the workstation (MMWP VE31A, Siemens Syngo) for post-processing. First, the conventional CT images, including the axial images and the reconstructed MPR, MIP, and VR, were used for evaluation and measurement. The evaluation indicators were as follows:

A: Single-line type or multiple-line type endoluminal intimal flaps in the axial or MPR images near the proximal entry tear;
B: Type I with a large false lumen and type II with a large true lumen based on the lumen area separated by an intimal flap;
C: Three dissection direction types (anterograde, retrograde, and bidirectional) of the entry tear;
D: Type I of tear with artifacts that may interfere with the display of the entry tear, and type II without artifacts.

In addition, we observed whether the dissection extended into the neighboring structures. The CT attenuation values (in HU) of the true and false lumens near an entry tear (within 2 cm) were also measured. Second, the VIE images of the torn inlet and outlet as well as that of the adjacent intimal flap were generated in the true lumen in most cases using the workstation because endovascular repair was typically conducted in the true lumen. During VIE imaging, an appropriate threshold was progressively changed from 0 HU to 500 HU to obtain excellent views, which might clearly show the entry tears and intimal flaps with fewer artifacts. Agreement was established when at least two observers agreed on the appropriate threshold, which clearly displayed the images for diagnosis. The evaluation indicators of VIE were as follows: A, three (round, slit-shaped, and irregular) types of entry tear shape; B, four (sheetlike, tubular, wavelike, and irregular) types of intimal flap shape; C, three classifications of the display quality of intimal tears, including grade 1 with optimum visualization of the tear, grade 2 with satisfactory visualization (which requires a slight adjustment of the threshold), and grade 3 with available visualization (which requires considerable adjustment). VIE was also used to visualize the adjacent intraluminal structures, neighboring aneurysm, transmural ulceration, and small torn flaps and their spatial relationships. The observation and measurement of all the images were performed based on the consensus between two radiologists with experience in cardiovascular radiology. The radiologists were blinded to the clinical data and the study objective.

### Statistical Analysis

Statistical analysis was performed using the SPSS software package version 17. The measured CT attenuation values were expressed as means ± SD. One-way analysis of variance with a confidence level of 95% was used to compare three classifications of the evaluated display quality of entry tears and flaps after the homogeneity of the variance test. The relationships of the evaluation indicators with VIE and other data were analyzed via the chi-squared (*x*^2^) test. A *P* value of less than 0.05 was considered statistically significant.

## Results

### Imaging Findings of the Conventional CT

**Fig 1:**
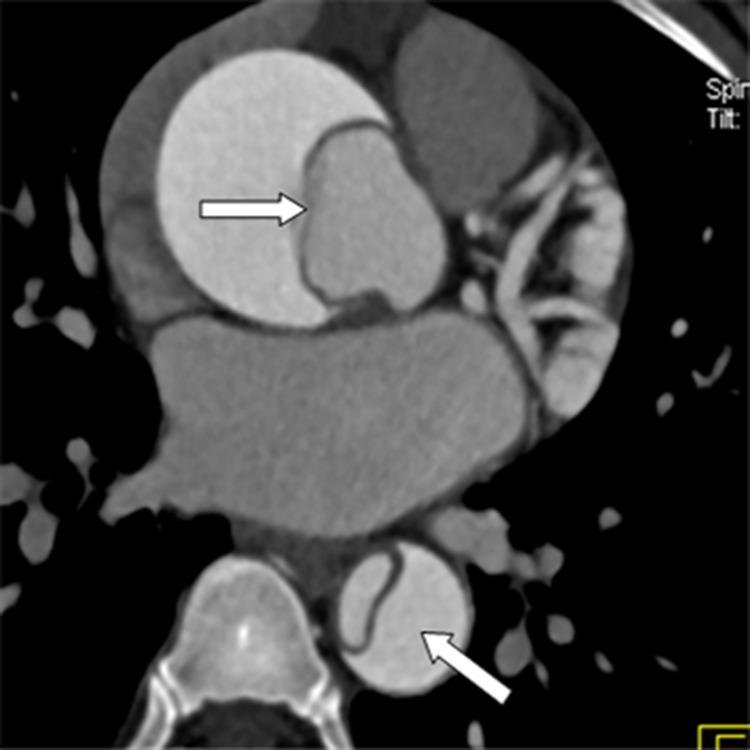
Single-line type of the intimal flap on an axial CT image.

**Fig 2:**
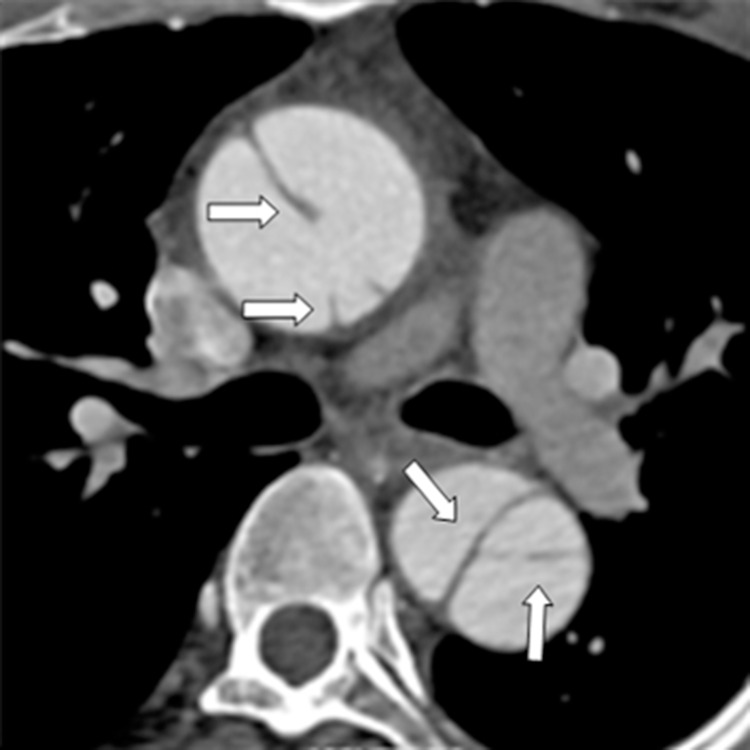
Multiple-line type of the intimal flap on an axial CT image.

The conventional CT revealed AD with Stanford type A in 46 patients and type B in 38 patients. No entry tear was found in 5 patients because of the neighboring thromboses. Accordingly, we identified 92 entry tears in the remaining 79 patients, 6 of whom had 2 entry tears, and 1 had 3. In addition, 43 torn outlets of the intimal flap were revealed in 32 patients, 6 of whom had 2 outlets, 2 had 3, and the remaining patients had one each. The locations of the proximal entry tears and torn outlets of the flap are shown in Table 1 (Figs 1 and 2), and several evaluation indicators of AD are presented in Table 2. The artifacts near 16 entry tears include the streak artifacts of the contrast medium in the left brachiocephalic vein or in the superior vena cava in 7, and the aortic motion artifacts in 9. The involved vessels by the tear of AD are also shown in Table 3. Neighboring aneurysms are identified in 7, whereas transmural ulcers are found in 11.

**Table 1:** Locations of the proximal entry tears and torn outlets of the intimal flap shown on the conventional CT.

Locations	Proximal entry tears n(%) 92	Torn outlets n(%) 43
**Ascending aorta**	34(37.0)	3(6.98)
**Aortic arch**	33(35.9)	9(20.9)
**Descending thoracic aorta**	7(7.61)	2(4.56)
**Abdominal aorta**	17(18.5)	17(39.5)
**Innominate artery**	1(1.09)	-
**Iliac artery**	-	12(27.9)

**Table 2:** Evaluation indicators of AD on the conventional CT.

	n (%) 92
**Intimal flaps near the entry tear**	
**Single-line type (Fig 1)**	32 (34.8)
**Multiple-line type (Fig 2)**	60 (65.2)
**Lumen area**	
**Type I with a large false lumen**	51 (55.4)
**Type II with a large true lumen**	41 (44.6)
**Artifact types of tear**	
**Type I with artifacts**	16 (17.4)
**Type II without artifacts**	76 (82.6)
**Dissection direction types of the entrance tear**	
**Anterograde**	37 (40.2)
**Retrograde**	43 (46.7)
**Bidirectional**	12(13.1)

**Table 3:** Involved vessels by the tear of AD on the conventional CT.

Involved vessels	n (%) 60
Aortic root*	31 (51.7)
**Innominate artery**	4 (6.67)
**Left subclavian artery**	4(6.67)
**Left common carotid artery**	3 (5.00)
**Three supra-aortic vessels**	2 (3.33)
**Renal artery**	9 (15.0)
**Celiac trunk**	3 (5.00)
**Superior mesenteric artery**	2 (3.33)
**Renal artery and celiac trunk**	2 (3.33)

* Involvement of the tear in coronary arterial ostia in 8.

### Visualization of the Intimal Tear on VIE

**Fig 3:**
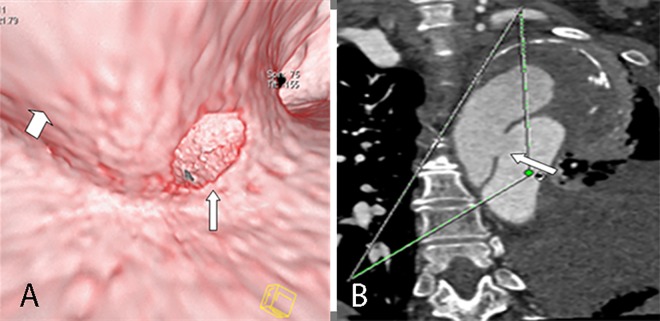
Round entry tear on VIE and MPR. VIE (A) shows the entire round entry tear (narrow arrow) on the intimal flap (wide arrow) viewed from the true lumen of a Stanford type A dissection. The false lumen exhibits “windsock” appearance. The MPR (B) displays only a portion.

**Fig 4:**
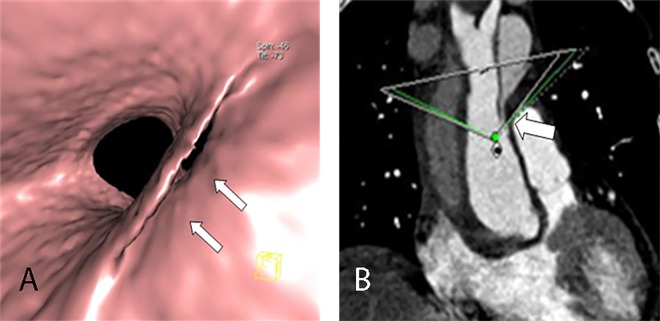
Slit-shaped entry tear on VIE and MPR. VIE (A) shows the entire slit-shaped entry tear (arrow) on the aortic wall viewed from the true lumen. The MPR (B) displays only a portion.

**Fig 5:**
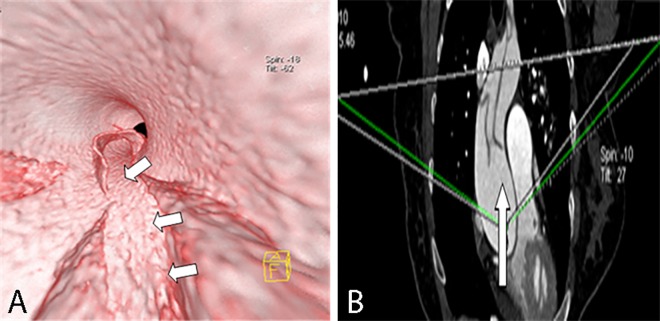
Irregular entry tear on VIE and MPR. VIE (A) shows a long irregular entry tear (arrow) on the intimal flap of a Stanford type A dissection viewed from the true lumen. The MPR (B) cannot show the actual configurations of the tear and flap.

When the imaging findings of the conventional CT was used as the standard, 95.7% (88/92) of the entry tears and 90.7% (39/43) of the torn outlets were displayed well on VIE. However, other tears were not displayed because of calcifications, contrast artifacts, and low signal-to-noise ratios. The 3D configurations of entry tears on VIE were round in 26 (29.5%, Fig 3), slit-shaped in 9 (10.2%, Fig 4), and irregular in 53 (60.2%, Fig 5). Among 58 entry tears with a multiple-line type flap on the conventional CT, 41 (70.7%) were irregular in shape on VIE (Fig 5). Meanwhile, among 30 entry tears with a single-line type flap, 17 (56.7%) were round or slit-shaped on VIE (Fig 3), which demonstrated a significant difference (*P =* 0.041, *x*^2^ *=* 6.409). However, the relationships of the three types of entry tear shape on VIE with other evaluation indicators were not found (*P* > 0.05).

### Visualization of the Intimal Flap on VIE

**Fig 6:**
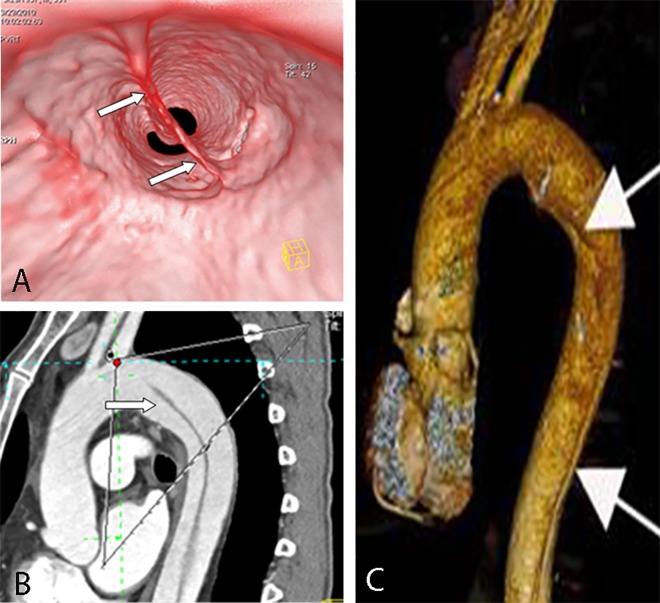
Sheetlike intimal flap on VIE, MPR, and VR. VIE (A) shows the entire sheetlike intimal flap (arrow) of a Stanford type B dissection. MPR (B) and VR (C) only show the flap as a line.

**Fig 7:**
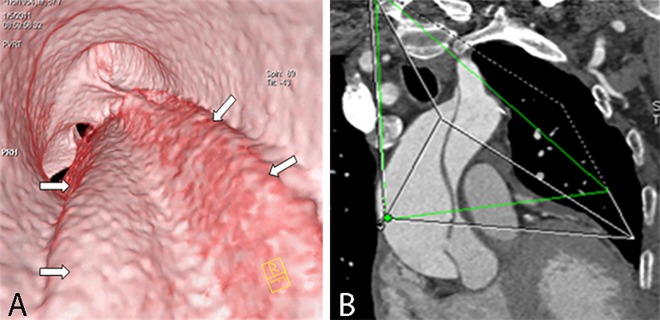
Wavelike intimal flap on VIE and MPR. VIE (A) shows the entire wavelike intimal flap (arrow) viewed from the true lumen (A) and involvement into the edge of three branches. MPR (B) shows only a portion.

**Fig 8:**
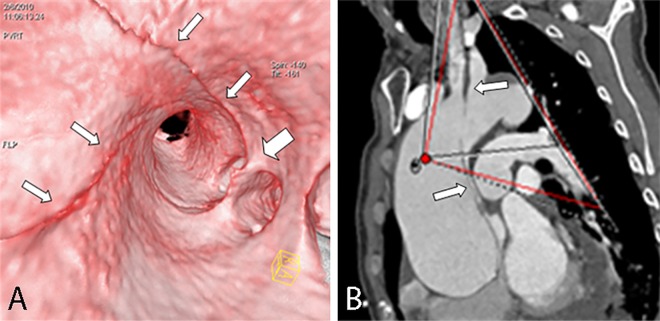
Two spiral tears of an intimal flap on VIE and MPR. VIE (A) shows two spiral tears of an intimal flap (narrow arrow) viewed from the true lumen; one of the spiral tears involves the innominate artery (wide narrow). MPR (B) cannot show the number and the spiral shape of the flap.

**Fig 9:**
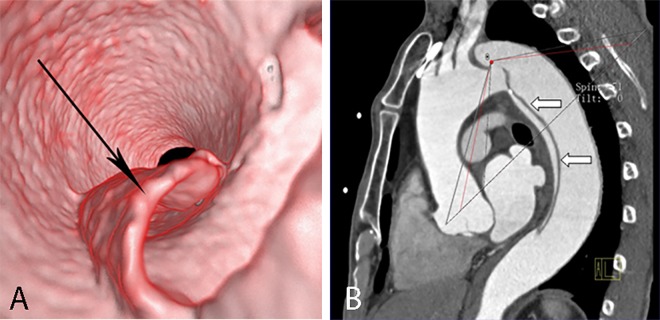
Spiral intimal flap on VIE and MPR. VIE (A) shows the entire spiral intimal flap (arrow) viewed from the true lumen, thereby providing a more accurate image than MPR (B).

**Fig 10:**
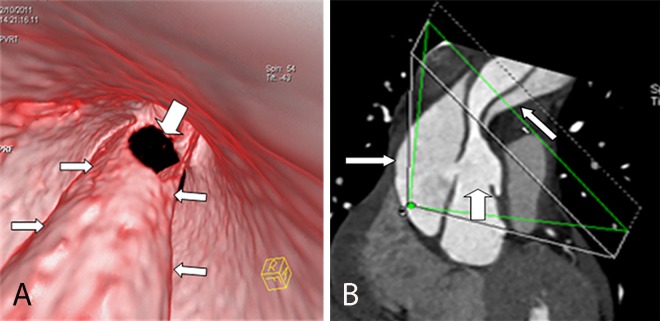
Intimointimal intussusception on VIE and MPR. VIE (A) shows the true lumen (narrow arrow) of the intimointimal intussusception appearing as a “windsock” with a round entry tear (wide arrow) in the flap, thereby providing a more accurate configuration and spatial relationship than MPR (B) with a multiple-line type flap (the true lumen, wide arrow) located between the false lumen (narrow arrow).

**Fig 11:**
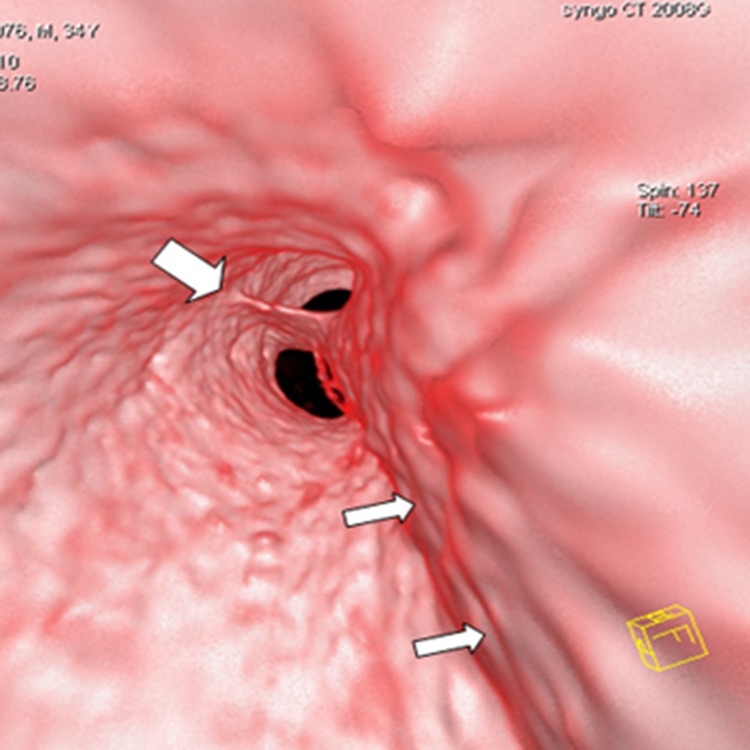
Multiple intimal flaps on VIE and axial CT. VIE shows three channels, two of which are separated by an intimal flap (wide arrow), and the third flap lies on the lateral side (narrow arrow). The axial CT (Fig 2) cannot show the entire shape and spatial relationship of the flaps, which appear as a “Mercedes-Benz sign” in the descending aorta.

**Fig 12:**
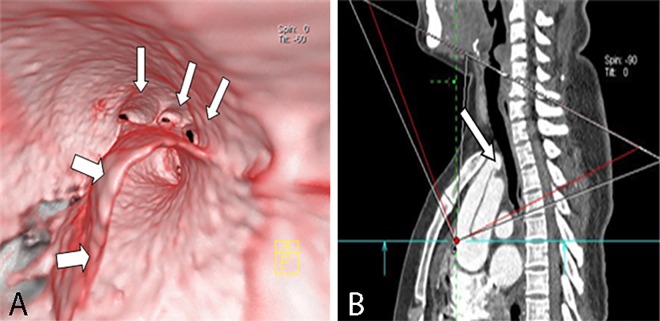
Involvement of a torn flap in the branches on VIE and MPR. VIE (A) shows complete involvement of the torn flap in three aortic branches and their spatial relationships (narrow arrow) with a more real and accurate visualization and location than MPR (B).

**Fig 13:**
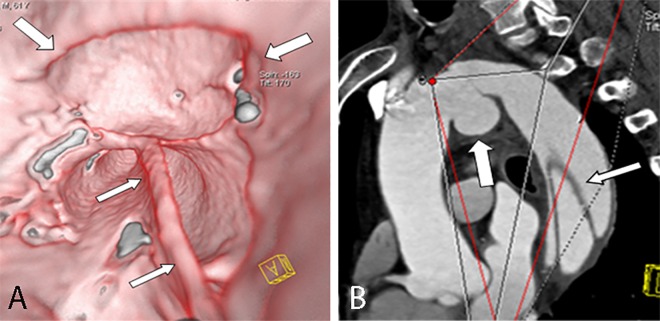
Aneurysm on VIE and MPR. VIE (A) shows a complete aneurysm (wide arrow) and a torn intimal flap (narrow arrow) derived from the edge of the aneurysm, thereby providing a more accurate shape and spatial relationships than MPR (B).

**Fig 14:**
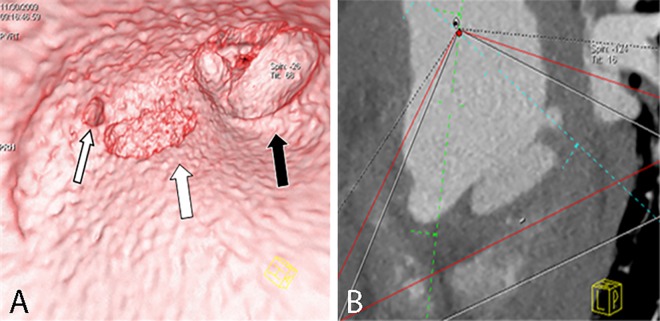
Transmural ulcerations on VIE and MPR. VIE (A) shows the complete shapes of two transmural ulcerations (wide white and black arrow) and the spatial relationship between the ulceration and an ostium of the coronary artery (narrow white arrow), thereby providing a more accurate visualization than MPR (B).

**Fig 15:**
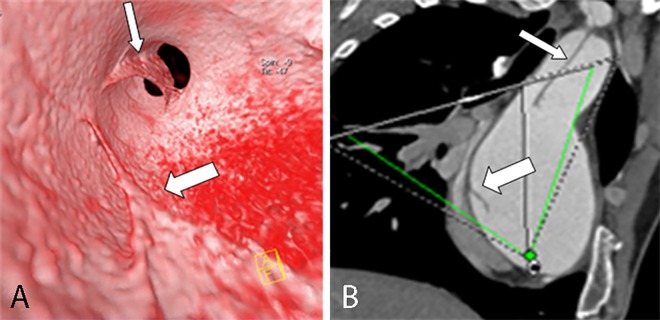
Two spirally torn flaps on VIE and MPR. VIE (A) shows a more accurate spatial relationship and configuration of the two spirally torn flaps than MPR (B).

The 3D configurations of the intimal flaps were displayed well on VIE with 4 types of shape: sheetlike in 34 (Fig 6), tubular in 34 (Fig 3), wavelike in 13 (Fig 7), and irregular in 7 (Fig 5). Among these configurations, a spirally torn flap was observed in 38 (Figs 8 and 9). The 3D “windsock” appearance of the false lumen in 49 (Figs 3, 5, 7 and 9) and that of true lumen in the intimointimal intussusception in 6 were observed (Fig 10). As for the “Mercedes-Benz sign” of the flap on the axial CT images of 5 patients, the 3D three-channel dissection was seen clearly on VIE (Figs 2 and 11). In addition, among the 58 flaps with a multiple-line type on the conventional CT, 50 (86.2%) were tubular, wavy, or irregular in shape on VIE. Meanwhile, among the 30 flaps with a single-line type, 26 (86.7%) had a thin sheetlike shape on VIE, which exhibited a significant difference (*x*^2^ = 44.491, *P* = 0.000). Moreover, the spatial relationship of the torn flap and adjacent aortic branches were shown well on VIE (Fig 12). The figure also illustrates eight involved coronary arteries and 13 involved supra-aortic vessels. Among 16 involved branches of abdominal aorta on the conventional CT, 11 were depicted well on VIE, whereas the other 5 were not displayed clearly because of the low signal-to-noise ratio or low contrast density. VIE also presented an excellent 3D view of aneurysm (Fig 13), transmural ulceration (Fig 14), and small torn flap (Fig 15).

### Factors That Influence the Display of the Intimal Tear on VIE

For the display quality of the tears on VIE, 63 (71.6%), 17 (19.3%), and 8 (9.1%) tears of grades 1–3 were respectively found. If the CT attenuation values in the false and true lumens around the entry tear decreased, then the display quality of VIE would decrease, which exhibited a significant difference in the CT values among the three grades (*P* < 0.01, Table 4). In addition, the poor display of the tears on VIE was significantly related to nearby artifacts (*x*^2^ = 27.719, *P* = 0.00). However, the relationships of the display quality with Stanford classification or with other evaluation indicators were not found (*P* > 0.05).

**Table 4:** Difference in CT attenuation values among three grades of the display quality of entry tears on VIE.

Location	Grades of the display quality of entry tears on VIE*			*F*(*P*)
	Grade 1 (HU)	Grade 2 (HU)	Grade 3 (HU)	
**True lumen**	324.22 ± 86.99	276.57 ± 75.71	212.83 ± 54.03	6.420 (0.003)
**False lumen**	310.26 ± 78.35	264.86 ± 74.22	216.84 ± 63.28	5.790 (0.005)

* Grades of the display quality of the entry tears on VIE: Grade 1, optimum visualization; Grade 2, satisfactory visualization with a slight adjustment; and Grade 3, available visualization with a considerable adjustment.

## Discussion

AD is an acute life-threatening emergency condition that frequently has a fatal outcome [16]. Early diagnosis and treatment are essential to improve prognosis [8]. CTA is considered as the first-line tool for a rapid and reliable diagnosis, with a sensitivity of 99% and a specificity of 100% [17–19]. With the increasing use of endovascular stents in the treatment of the disease, conventional CTA with 2D reformation and 3D rendering plays an essential role in the imaging of dissection, thereby providing necessary pre- and post-stent placement information [4]. However, these techniques also exhibit certain limitations. For example, although VR may show the 3D configuration of the dissection, this approach is limited in terms of providing a precise and direct visualization of the intimal tear and flap. AD evaluation is typically based on the axial and MRP images; however, the images show only a partial intimal tear and flap rather than entire 3D view. MIP, a technique that highlights the highest attenuation voxels, is unable to discern overlapping structures, such as the intimal tear, flap, and the surrounding contrast medium because of its lack of depth perception [10]. Thus, we cannot obtain the 3D structures of intimal tear and flap based on these conventional CT techniques. A marked difference in imaging manifestations is observed between the 3D view and the conventional CT methods, particularly for complicated cases. In addition, currently used conventional CT techniques cannot fulfill the requirement of accurate and precise treatment planning.

By contrast, VIE is performed by applying volume-rendered thresholds and spatial rendering to generate endoluminal views. VIE provides an excellent 3D view to clearly show the entry tear and intimal flap that may overcome the deficiency of the conventional CT [11,13,14]. To date, however, VIE has not yet been widely used in clinical practice. This approach has been previously reported to be available for assessing endovascular stent grafts for AD [10,13,14,20,21]. In addition, the literature regarding the preoperational visualization of the intimal structure of AD remains limited [10,15,20]. Our results indicated a good complete display of the torn inlet and outlet at 95.7% and 90.7%, with a satisfactory display of the entry tear (grades 1 and 2 display quality) at 90.9%. Therefore, VIE is an indispensable method for visualizing the 3D structures of intimal tears and flaps, which enhances understanding on endovascular abnormalities and provides additional details for assessment, treatment, and follow-up. Our VIE showed that most entry tears (60.2%) were irregular in shape, whereas 61.4% of the flaps were tubular, wavy, or irregular, thereby indicating that VIE may perform a more important role in visualizing complicated endovascular changes. On VIE, for example, the real 3D configuration of the “Mercedes-Benz sign” appearing on the axial CT is a three-channel flap [8], and VIE also displays the true shape of intimointimal intussusception, which is an unusual manifestation of AD in which the inner true lumen is wrapped around by the false lumen [10]. Hence, readers should be cautious when interpreting the shapes of tears and flaps that are only based on the conventional CT. Moreover, VIE can perform more important functions in the direct visualization of the real spatial relationship between the intimal involvement of the tear and adjacent structures, as well as in displaying accompanying changes, such as spiral tear, aneurysm, transmural ulceration, and other small torn flaps. Our results are consistent with the report of Sun [10].

We also investigated why certain entry tears were not shown well on VIE for a portion of the patient population. Our results show that the display quality of the intimal tear worsens with the decrease in CT values, thereby indicating that the concentration of the contrast agent within the lumen is a critical factor that affects VIE display. Therefore, the optimization of the scanning protocols becomes more vital, including appropriately increasing the amount of the contrast agent and the injection speed to ensure high concentration within the lumen. By contrast, we also found a close relationship between the display quality of VIE and the surrounding artifacts, which led to a decrease in lumen transparency that affected VIE imaging quality. Adequate and uniform aortic enhancement throughout the entire period of image acquisition is highly desirable to obtain an excellent view of tears and flaps on VIE [22]. In addition, the artifacts can result in blurred tear and flap outlines [22]. Given that most problematic streak artifacts originate from the enhanced left brachiocephalic vein and the superior vena cava, different strategies, such as the use of diluted contrast materials, the injection of contrast materials into a right antecubital vein or the lower extremity vein, and scan acquisition in a caudocranial direction, have been used to minimize artifacts [23,24]. Other factors, such as the selection of the bolus-timing software for optimization of the contrast delivery timing, the use of ECG-gating, and a reasonable reconstruction phase, are considered to reduce artifacts.

The VIE technique has several limitations. First, the distortion of images on VIE is difficult to avoid. Therefore, certain differences in the shape of the intimal tears and flaps between the actual morphology and VIE manifestations are observed. Second, the shape of tears and flaps may change alternately because of the fluctuating pressure during the cardiac cycle. Third, the precise measurements of the range and scope of AD cannot be performed via VIE imaging. Fourth, the radiation dose in this study is higher because of the larger scanning range, which may be minimized by decreasing the scanning range of the involved vessel based on the results of sonography and by using an advanced CT scanner. Moreover, our VIE results also lack comparison with surgical specimens of the involved aorta.

## Conclusions

CT VIE demonstrates a unique value in the visualization of 3D intravascular configurations of the intimal tears and flaps of AD, particularly for complicated changes. VIE highlights more detailed information to facilitate the adaptation of endovascular therapy. This technique should be regarded as a necessary tool for assessment and strategy planning to improve AD prognosis.
